# Impairments of cerebellar structure and function in a zebrafish KO of neuropsychiatric risk gene *znf536*

**DOI:** 10.1038/s41398-024-02806-1

**Published:** 2024-02-08

**Authors:** Tae-Yoon Kim, Arkaprava Roychaudhury, Hyun-Taek Kim, Tae-Ik Choi, Seung Tae Baek, Summer B. Thyme, Cheol-Hee Kim

**Affiliations:** 1 Department of Biology, Chungnam National University, Daejeon, 34134 South Korea; 2 Soonchunhyang Institute of Medi-bio Science (SIMS), Soonchunhyang University, Cheonan, 31151 South Korea; 3 Department of Life Sciences, Pohang University of Science and Technology (POSTECH), Pohang, 37673 South Korea; 4 Department of Neurobiology, University of Alabama at Birmingham, Birmingham, AL USA; 5 Present Address: Department of Biochemistry and Molecular Biotechnology, UMass Chan Medical School, Worcester, MA USA

**Keywords:** Molecular neuroscience, Genetics

## Abstract

Genetic variants in *ZNF536* contribute to the risk for neuropsychiatric disorders such as schizophrenia, autism, and others. The role of this putative transcriptional repressor in brain development and function is, however, largely unknown. We generated *znf536* knockout (KO) zebrafish and studied their behavior, brain anatomy, and brain function. Larval KO zebrafish showed a reduced ability to compete for food, resulting in decreased total body length and size. This phenotype can be rescued by segregating the homozygous KO larvae from their wild-type and heterozygous siblings, enabling studies of adult homozygous KO animals. In adult KO zebrafish, we observed significant reductions in anxiety-like behavior and social interaction. These *znf536* KO zebrafish have decreased cerebellar volume, corresponding to decreased populations of specific neuronal cells, especially in the valvular cerebelli (Va). Finally, using a *Tg[mbp:mgfp]* line, we identified a previously undetected myelin structure located bilaterally within the Va, which also displayed a reduction in volume and disorganization in KO zebrafish. These findings indicate an important role for *ZNF536* in brain development and implicate the cerebellum in the pathophysiology of neuropsychiatric disorders.

## Introduction

Schizophrenia is a neuropsychiatric disorder with a lifetime risk of 1%, much of which is attributable to genetic factors [1, 2]. This genetic risk includes common variants [3], copy number variants [4], and rare protein-coding variants [5]. Although therapeutic development for schizophrenia has advanced over the past few decades, efficacy remains low [6, 7] due to genetic contributions to underlying pathophysiology remaining largely unknown [4].

Genomic studies have implicated the gene *ZNF536* as a candidate risk factor for neuropsychiatric disorders, particularly schizophrenia. Single-nucleotide polymorphisms (SNPs) associated with schizophrenia were identified in the *ZNF536* gene, mainly located within introns 2 and 3 (Fig. S1) [2]. The impact of these SNPs on *ZNF536* expression level or splicing is currently unknown. However, rare protein-truncating variants in *ZNF536* have been found in individuals with autism spectrum disorder [8, 9]. Weak associations with bipolar disorder, major depressive disorder, and ADHD have also been reported [10–12].

*ZNF536* encodes a putative transcriptional repressor containing ten adjacent zinc finger domains, which are highly conserved in vertebrates [13]. Although it was reported to negatively regulate neuronal differentiation by retinoic acid/RAR-induced transcriptional activity [13], its physiological function is largely unknown. Previously, a large screen of genes linked to neuropsychiatric disorders discovered abnormalities in brain structure and function in *znf536* knockout (KO) zebrafish larvae [14]. However, this study was limited to the larval stage and did not include extensive characterizations of individual gene KO mutants.

Zebrafish are a powerful model organism for the study of genes involved in neuropsychiatric disorders [15]. They are a well-characterized model for studying autism spectrum disorders (ASDs) [16–18] and for validating the function of human candidate genes for neurodevelopmental disorders [19–22]. Moreover, major human brain regions are anatomically and functionally conserved in fish, including the amygdala, habenula and cerebellum [23, 24].

In this study, we aimed to determine how *ZNF536* contributes to the development of neuropsychiatric disorders by studying its role in the adult zebrafish brain. We characterized the behavior, neuroanatomy, and neural function of *znf536* KO zebrafish.

## Materials and Methods

### Zebrafish Husbandry

A closed line of wild-type zebrafish (*Danio rerio*) maintained in our animal facility were reared under standard conditions with a 14:10-h light: dark cycle. Harvested embryos were kept in egg water (5 mM NaCl, 0.17 mM KCl, 0.33 mM CaCl_2_, 0.33 mM MgSO_4_,10-5% Methylene Blue) at 28.5 °C. Both wild-type and *mbp:mgfp* transgenic lines were obtained from the Zebrafish Center for Disease Modeling (ZCDM), South Korea. The age of the animals is reported in the figure legends. Adult male zebrafish or larvae without a defined sex were used. All zebrafish experiments were approved by the Institutional Animal Care and Use Committees (IACUC) of Chungnam National University (202012A-CNU-170).

### Generation of *znf536* KO zebrafish model using CRISPR/Cas9

**Fig. 1:**
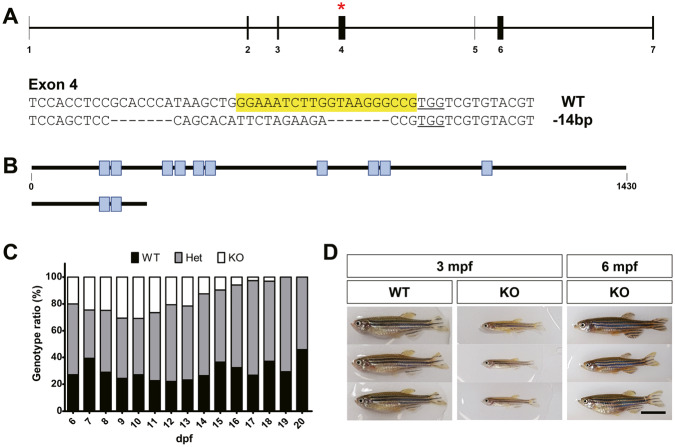
Generation of *znf536* KO zebrafish and growth phenotype analysis. **A** Generation of the *znf536* KO line. Red asterisk (upper panel) and yellow box (lower panel) indicate the sgRNA target region. **B** Schematic representation of predicted proteins in the WT and KO. Blue boxes indicate the zinc finger C2H2 domains. **C** Genotype ratio for larval stage zebrafish from heterozygous incrosses (6–20 dpf) (*n* = 1433). **D** Representative images of *znf536* WT and KO zebrafish at 3 and 6 mpf adult stages. *n* = 14 for WT, *n* = 44 for Het, and *n* = 9 for KO (3 mpf); *n* = 9 for KO (6 mpf). Scale bar: 10 mm.

We used CRISPR/Cas9 technology to generate zebrafish with a knockout of the *znf536* gene. A sgRNA targeting exon 4 of *znf536* (GRCz11, ENSDARG00000103648) was selected using CRISPR DESIGN (http://crispr.mit.edu/). To identify somatic mutations, genomic DNA was isolated using the HotSHOT method, and T7 endonuclease I (NEB Ipswich, Massachusetts, USA) digestion was performed on a 175 base-pair fragment amplified using the following primer pair: 5′-CGATTCCGCTTCAACAGCATT-3′ (F) and 5′-TTTCCTCACGTACACGACCA-3′ (R). The identified founder zebrafish were crossed with wild-type, and germline transmission resulted in the propagation of *znf536*^ck109a^ KO line with 14-bp deletion (31-bp deletion with 17-bp insertion) (Fig. 1A).

### Whole-mount in situ hybridization

Anti-sense digoxigenin-labeled RNA probes (Table S1) were synthesized with in vitro transcription kits according to the manufacturer’s protocol. Embryos were fixed overnight in the 4% Paraformaldehyde (PFA) (Sigma-Aldrich) in PBS (pH 7.4) at 4 °C. Adult zebrafish were fixed in the same conditions for 3 days prior to brain isolation. All samples were washed with DEPC (Diethylpyrocarbonate, Sigma)-PBST(PBS with 0.1% Tween 20) and stored in methanol at −20 °C. Whole-mount in situ hybridization was performed as previously described [25]. WISH images were taken using MZ16FA bright microscope (Leica Microsystems, Germany) in 90% glycerol (in PBST) or BB solution (2:1 ratio of benzyl benzoate and benzyl alcohol mixture) as the mounting solution. All images were processed using GIMP (Charlotte, North Carolina, USA) software.

### Food competition test

To assess food competition, we provided zebrafish with rotifer (*Brachionus calyciflorus*), a moving live food. Before the test at 14 dpf, zebrafish larvae were adapted to rotifers by feeding them three times a day from 5 to 13 dpf. During the test, twenty larvae were placed in a 60 mm petri dish with 50 rotifers for 10 min. Larvae were imaged with an MZ16FA brightfield microscope (Leica Microsystems, Germany), and the number of rotifers within the digestive tract in the individuals was counted; each larva was subsequently genotyped.

### Shoaling bowl assay

The shoaling bowl assay was conducted as previously described [16]. To assess whether *znf536* KO zebrafish show modified shoaling behavior, we placed three adult animals of the same genotype in a bowl (upper half diameter, 33 cm; bottom diameter, 24 cm; height, 11 cm; and water depth, 3.2 cm) and acquired a top-view video for 15 min with a Sony HDR-CX190 camera. The distance between individual fish was measured in video frames taken every 1 s for 5–10 min using the ImageJ program (imagej.nih.gov) (NIH, Bethesda, Maryland, USA).

### Novel tank assay

The novel tank assay was conducted as previously described [17]. Adult wild-type, heterozygous, and homozygous KO zebrafish were placed in a tank (24 × 15 × 15 cm) covered with non-transparent white paper on the back and sides. A total of 15 min of video was taken using a video camera (Sony, HDR-CX190) from the lateral side. All behavioral experiments were conducted between 1:00 pm and 5:00 pm. The acquired video was analyzed using EthoVision XT 17 (Noldus, The Netherlands).

### Social interaction assay

The social interaction assay was conducted as previously described [16]. The behavior tank (24 × 12.5 × 12.5 cm), covered on three sides with non-transparent material and open on one side, was filled with over three liters of system water. The tank was divided into two sections with a transparent acrylic partition. One section of the chamber was designated for the cue fish, whereas the other part of the chamber was assigned to the tester fish. The tester section was divided into four chambers (very close, close, far, and very far). The very close zone was designated as the very close/nearest zone to the cue fish. The very close and very far zones were 2 cm wide, while the close and far zones were 7 cm wide. All behavioral experiments were conducted between 1:00 pm and 5:00 pm. Three adult wild-type zebrafish were used as the cue fish, and single *znf536* KO and control siblings were used as the tester fish. A total of 20 min of video was taken from the lateral side using a SONY HDR-CX405 camera. Videos were analyzed using EthoVision XT 17 (Noldus, The Netherlands).

### Acoustic startle response assay

To test the acoustic startle response of adult zebrafish, we built an apparatus as previously described [26]. A CMOS webcam HF1316 (SUYIN) and stereo speakers were isolated from a laptop. The speakers were connected to a digital power amp module PAM8403 for compatibility with 5 V USB. The body of a stereo microscope (Leica) was used to support each part, and a light source was used as the backlight. A single adult zebrafish was placed in a 100 mm plant culture dish (SPL, Korea) with system water (Fig. S4A). We used OBS Studio (OBS Project, https://obsproject.com/), an open-source recording and streaming software for video recording. Sound stimuli were introduced three times as a 1 kHz, 90 dB, 0.5-s sine wave in 1-min intervals, after adaptation for 5 min. The experiments were performed in an isolated space to minimize the impact of ambient noise on the zebrafish behavior. Videos were analyzed using EthoVision XT 17 (Noldus, the Netherlands).

### Adult brain tissue clearing and imaging

To observe the brain’s internal structure, we performed tissue clearing using the Binaree Tissue Clearing Starter’s Kit (Binaree, Daegu, Korea), according to the manufacturer’s protocol, with some modifications for zebrafish. Briefly, *mbp:mgfp* transgenic line background *znf536* KO adult fish were fixed in the 4% PFA (Sigma-Aldrich) in PBS (pH 7.4) for three days at 4 °C. Isolated brain samples were washed three times with PBS. The samples were incubated with 500 μl Starting Solution at 4 °C until they sank. Next, the samples were incubated with 500 μl Tissue Clearing Solutions in the hybridization incubator at 37 °C for one day and washed three times with distilled water. These two steps were repeated one more time. The transparent brain samples were mounted with a 700 μl Mounting & Storage Solution (Binaree, Daegu, Korea). The samples were imaged with a Zeiss LSM 880 confocal laser microscope (Carl Zeiss, Oberkochen, Germany).

### Histology

Paraffin sectioning and hematoxylin and eosin (H&E) staining of adult zebrafish brains were completed as previously described [16]. Briefly, adult zebrafish were fixed using 4% PFA (Sigma-Aldrich, St. Louis, Missouri, USA) in 1x PBS overnight. After fixation, the samples were dehydrated and stored in 70% EtOH at −20 °C. Paraffin embedding and sectioning were performed to obtain 7 µm sections for H&E staining. Tissues were imaged with an MZ16 brightfield stereo microscope (Leica Biosystems, Wetzlar, Germany). The relative size of valvular cerebelli (Va) of *znf536* KO zebrafish and WT zebrafish was measured by ImageJ analysis of the area of this region divided by the total area of the brain in the slice (Fig. S5B).

### Statistical analysis

For the adult behavior tests, the distance moved and zone duration times were automatically calculated by EthoVision XT 17 software. All statistical analyses were performed using SPSS 26 (IBM Corporation, USA) or Prism 10 (GraphPad, USA). Data was presented as mean ± standard error of the mean (S.E.M.). Statistical significance was determined by the Wilcoxon signed-rank test, Mann-Whitney test or Kruskal–Wallis test with post-hoc Dunn’s multiple comparisons test. The sample size was chosen based on past studies conducting the same experiments. No animals were excluded from the analysis, and the samples were not randomized or blinded.

## Results

### Generation of *znf536* KO zebrafish lines and analysis of abnormal growth

To characterize the spatiotemporal expression of *znf536* in larval and adult zebrafish, we performed whole-mount in situ hybridization with an anti-sense RNA probe. We observed robust expression in the nervous system from early somite stages (Fig. S2A). Similarly, in the adult brain, we observed regions of specific expression in the telencephalon, periventricular gray zone (PGZ) in the midbrain, and the cerebellum (Fig. S2B); however, *znf536* was not expressed in the olfactory bulb or optic tectum. To evaluate the role of *znf536* in neural function, we generated a *znf536* KO zebrafish line. We used CRISPR/Cas9-mediated mutagenesis to induce a 14-bp deletion within the fourth exon of *znf536* (Fig. 1A). The F1 embryos born from the outcross of the F0 were raised and genotyped, and a stable F2 line was generated. Targeted disruption of exon 4 is predicted to yield a truncated Znf536 protein missing multiple zinc finger C2H2 domains (Fig. 1B). The levels of *znf536* transcript were substantially reduced in *znf536* KO compared to WT adult brains, presumably from nonsense-mediated decay (Fig. S3).

We initially observed that no *znf536* KO zebrafish successfully reached adulthood. Thus, to check the survival rate, we genotyped several clutches daily from 6 to 20 dpf and analyzed the percentage of homozygous KO out of 1433 zebrafish larvae. We found that the number of *znf536* KO zebrafish consistently decreased after 14 dpf (Fig. 1C). Ultimately, we successfully obtained homozygous KO zebrafish at 3 months post-fertilization (mpf), but they were substantially smaller in size than their wild-type and heterozygous siblings. To enhance the growth of KO zebrafish, we isolated smaller fish from bigger siblings by 1 mpf and raised them separately in a 3-L tank, regularly feeding them with *Artemia salina*. When competition in the tank was reduced, *znf536* homozygous KO mutants recovered from the growth retardation by 6 mpf when compared to wild-type and heterozygous siblings (Fig. 1D). This approach was used to generate adult KO animals for all subsequent experiments. Taken together, we concluded that a cause of growth deficiency could be insufficient food availability during group foraging.

### *znf536* KO zebrafish have a reduced ability to compete for food

**Fig. 2:**
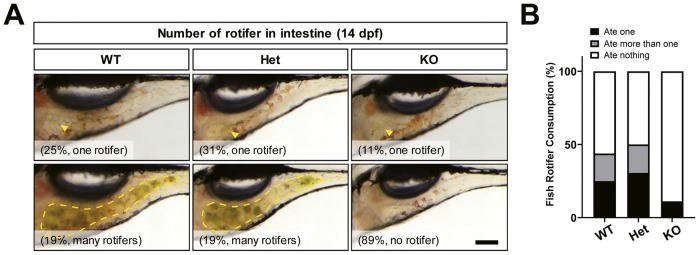
Food competition test. **A** Example image of rotifers in the intestine of zebrafish at 14 dpf. The arrowhead or dashed line indicates rotifers in the gastrointestinal tract. Scale bar: 200 µm. **B** Categorization of rotifer consumption by genotype. *n* = 16 for WT, *n* = 36 for Het, and *n* = 9 for KO.

To determine whether the cause of KO mutant growth deficiency was an inability to hunt effectively in a group context, we conducted a food competition assay. By 5 dpf, larval zebrafish could actively hunt for food and have a fully developed digestive tract [27]. The larvae were fed daily from 5–13 dpf, and the competition test was completed at 14 dpf by providing KO and sibling larvae with a limited number of rotifers for 10 min. We measured the quantity of consumed food by imaging the digestive tract of the transparent larvae (Fig. 2A). Over 80% of *znf536* KO larvae failed to consume any rotifers, whereas approximately 50% of wild-type and heterozygous siblings consumed at least one rotifer (Fig. 2B). Given this behavioral abnormality, we hypothesized that *znf536* KO zebrafish have differences in other behaviors and brain function.

### Anxiolytic behavior in *znf536* KO zebrafish

**Fig. 3:**
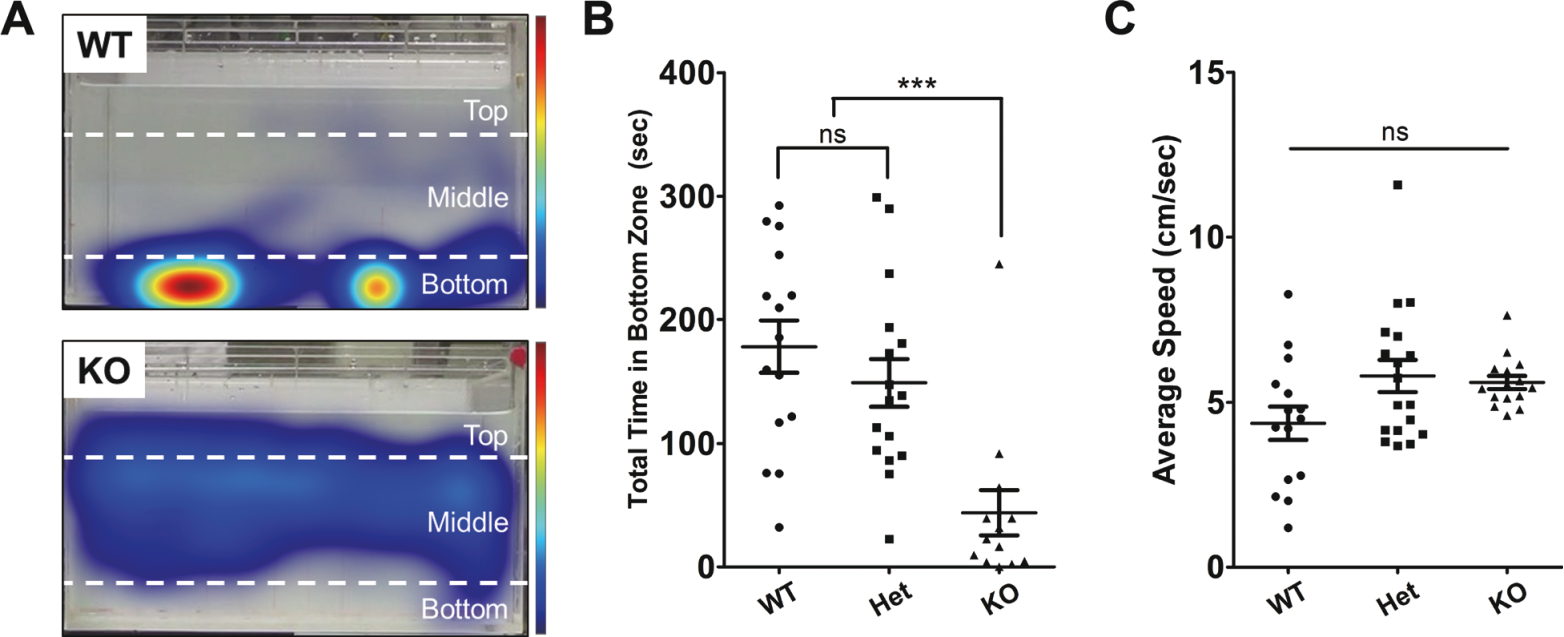
Decreased anxiety-like behavior in *znf536* KO zebrafish. **A** Representative heatmaps from the novel tank assay. The red color indicates increased dwell time and blue indicates decreased. **B** Quantified data for total time spent in the bottom zone. **C** Average speed of locomotion. *n* = 15 for WT, *n* = 16 for Het, and *n* = 13 for KO. Data was presented as mean ± standard error of the mean (S.E.M.). Statistical significance was determined by the Kruskal–Wallis tests with post-hoc Dunn’s multiple comparisons tests. ns, no significance; p*** < 0.001.

Extending our behavioral analysis to adult paradigms, we used the novel tank assay to measure anxiety-like behavior. This assay is comparable to the murine open-field test. When introduced to a new environment, zebrafish tend to stay at the bottom of the tank [28, 29]. In this study, we divided the tank into three zones (top, middle, and bottom) (Fig. 3A). We observed that *znf536* KO zebrafish spent significantly less time in the bottom zone while exploring the middle and top zones. In contrast, wild-type siblings spent more time in the bottom zone (Fig. 3B). We did not observe a significant difference in the average speed of locomotion between wild-type and KO zebrafish (Fig. 3C). In an acoustic startle response assay, *znf536* KO zebrafish showed reduced movement following a sound stimulus compared to WT (Fig. S4), implying a possible impairment in their response to aversive stimuli [30, 31]. These results indicated that *znf536* KO zebrafish had reduced anxiety-like (anxiolytic) behavior and that this phenotype was not due to overall differences in their locomotion.

### Impaired social interaction in *znf536* KO zebrafish

**Fig. 4:**
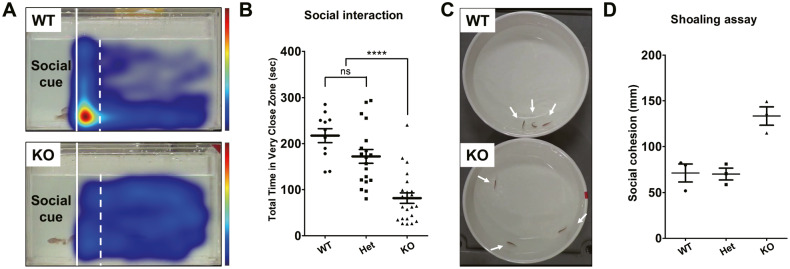
Impairments of social interaction in *znf536* KO zebrafish. **A** Representative heatmap of the social interaction assay. The red color indicates increased dwell time and blue indicates decreased. Solid lines indicate a transparent divider and dashed lines indicate the very close zone (2 cm from the social cue region). **B** Quantified data for duration within the very close zone. *n* = 11 for WT, *n* = 19 for Het, and *n* = 23 for KO. Data was presented as mean ± standard error of the mean (S.E.M.). Statistical significance was determined by Kruskal–Wallis test with post-hoc Dunn’s multiple comparisons test. ns, no significance; p**** < 0.0001. **C** Representative image from the shoaling assay. Individual adult fish were pointed by arrows. **D** Quantified data for the average of the inter-fish distance of the shoaling assay. The test was performed 3 times. For each trial, 3 fish of each genotype were used.

Fishes interact with each other in the form of shoaling [32–35]. Zebrafish exhibit highly sensitive shoaling behavior, which emerges after approximately 21 dpf [36–38]. The distance between individual fish is a proxy for social interaction [17, 39, 40]. First, we assessed the interaction of a single adult *znf536* KO zebrafish with three stimulus fish through a transparent divider. We found that wild-type siblings spent significantly more time in the very close (2 cm) zone interacting with the social cue fish than did their *znf536* KO siblings (Fig. 4A, B). Second, we assessed social cohesion by measuring the distance between individual fish in a group setting. We used three siblings from each genotype per trial (Fig. 4C). We found that *znf536* KO zebrafish had reduced social interaction in a group compared to their wild-type siblings (Fig. 4C, D).

### Reduced size of cerebellar structures in *znf536* KO zebrafish

**Fig. 5:**
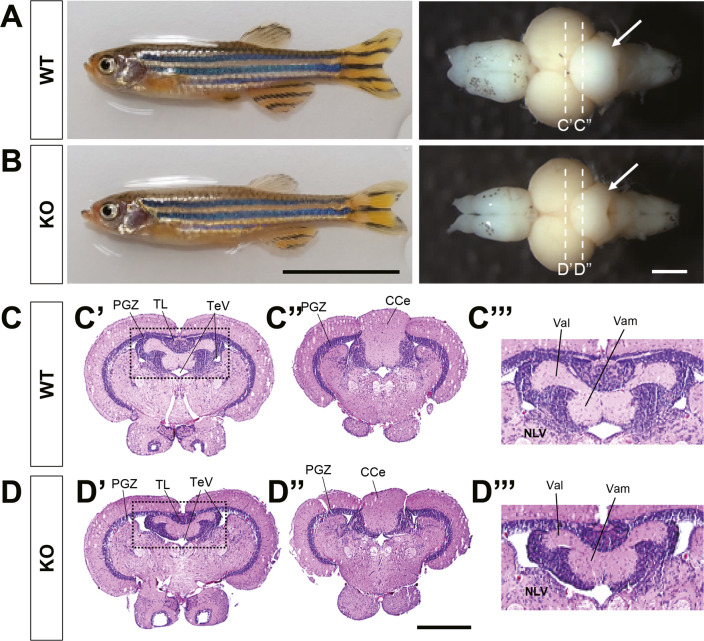
Anatomical and histological analysis of adult brains. **A**, **B** Representative images of *znf536* KO adult zebrafish at 11 mpf and their isolated brains. Arrows indicate the reduced size of the cerebellum in KO zebrafish. *n* = 7 for WT and *n* = 6 for KO. Scale bar: 10 mm (left panels) and 500 µm (right panels). **C**, **D** Brain section images at positions indicated by dashed lines in **A**, **B**. Serial sections were carefully examined to compare relative planes. **C”’**, **D”’** Magnification of marked areas in **C’**, **D’** (dashed box). ImageJ analysis **C’**, **D’** showed that the relative size of valvular cerebelli of *znf536* KO zebrafish (average 7.8% of the whole brain, *n* = 4) was reduced, compared to that of WT zebrafish (average 9.6%, *n* = 3). CCe corpus cerebelli, NLV nucleus lateralis valvulae, PGZ periventricular gray zone of optic tectum, TeV tectal ventricle, TL torus longitudinalis, Val lateral division of valvular cerebelli, Vam medial division of valvular cerebelli. Scale bar: 500 µm.

Since we observed numerous behavioral abnormalities in *znf536* KO zebrafish, we hypothesized defects in brain anatomy or function. We fixed adult animals of comparable body lengths and isolated brain tissue. The *znf536* KO zebrafish brains were of similar overall length but showed a reduced cerebellar size compared to WT siblings (Fig. 5A, B). H&E staining of paraffin sections revealed that the valvular cerebelli (Va), a part of the cerebellum in fish, had a smaller area in KO zebrafish. ImageJ analysis showed that the relative size of Va in the whole brain of *znf536* KO adult brain was reduced (*n* = 3 for WT, *n* = 4 for KO) (Fig. 5C, D”’ and Fig. S5).

### Reduced expression of neuronal markers and novel finding of myelinated neural circuits in the valvular cerebelli

**Fig. 6:**
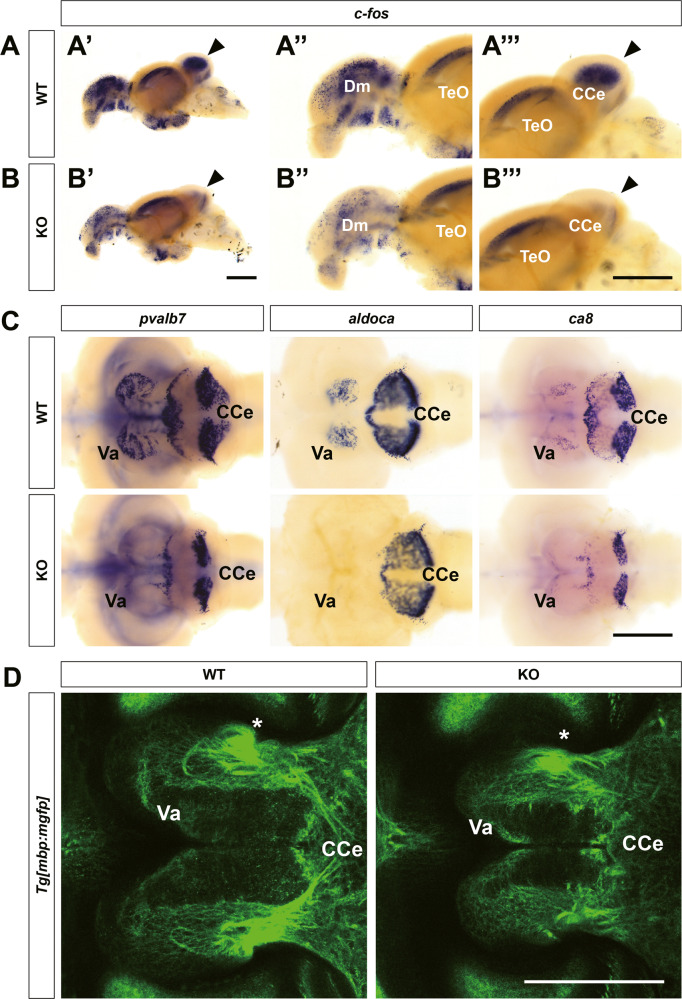
Defects in cerebellar structure and function in *znf536* KO zebrafish. **A**, **B”’** Reduced neuronal activity in KO zebrafish monitored by *c-fos* expression after novel tank assay, especially in the cerebellum (arrowheads). *n* = 3 for WT and *n* = 3 for KO. **C** Expression of Purkinje cell markers (*aldoca*, *pvalb7, ca8*) were specifically affected in the Va region. *n* = 3 for WT and *n* = 3 for KO for each marker. **D** Confocal images of myelinated bundles in *Tg[mbp:mgfp]::znf536* KO brain, focused in the Va region. A unique circular structure (asterisk) was identified within the Va. *n* = 2 for WT and *n* = 2 for KO. CCe corpus cerebelli, Dm medial zone of the dorsal telencephalic area, TeO optic tectum, Va valvular cerebelli. Scale bars: 500 µm.

To determine whether the observed anatomical changes led to functional deficits in the brain, we performed whole-mount in situ hybridization 30 min after the novel tank assay. While *c-fos* was expressed in both *znf536* KO and wild-type brains after this assay, there was substantially reduced expression in the telencephalon and cerebellum in KO brains (Fig. 6A, B and Fig. S6).

As the cerebellum of *znf536* KO zebrafish showed both anatomical and functional defects, we examined it more closely through marker gene analysis. The teleost cerebellum predominantly consists of the valvular cerebelli (Va), the corpus cerebelli (CCe), and the vestibular lobe consisting of the eminentia granularis (EG) and the lobus caudalis cerebelli (LCa) [41]. The Va and CCe consist of a three-layered structure, with the molecular layer (ML) being the outermost layer, followed by the Purkinje cell layer (PCL) and granule cell layer (GCL). At the early larval stage of 6 dpf, we observed a decrease in the structure of the Va in *znf536* KO zebrafish stained with anti-Synaptotagmin 2 (Znp-1), anti-Postsynaptic density-95 (MAGUK), or anti-Parvalbumin (Pvalb7) antibody [24] (Fig. S7). *Parvalbumin 7* (*pvalb7*) and *carbonic anhydrase 8* (*ca8*) are known to be expressed in the Purkinje cell layer [24]. Similarly, the promoter of *aldolase C, fructose-biphosphate, a* (*aldoca*) gene has been identified as a driver for Purkinje cell-specific gene expression [42]. Here, we saw a decrease in the population of these Purkinje cell marker-positive cells in the cerebellum; especially in the Va of adult *znf536* KO zebrafish when compared with wild-type siblings (Fig. 6C and S6). Similarly, we observed a decrease in the size of the expression region of *glutamate decarboxylase 1b* (*gad1b*) and *glutamate receptor, ionotropic, kainate 2* (*grik2*), especially in the cerebellum (Fig. S8). However, we could not detect dramatic changes in the expression of dopaminergic neuronal markers (*tyrosine hydroxylase 1, th1* and *tyrosine hydroxylase 2, th2*) or a stress-related marker (*corticotrophin-releasing hormone b, crhb*) [16, 17, 22, 43] (Fig. S9).

To examine cerebellar connectivity, we crossed *znf536* KO zebrafish with *Tg[mbp:mgfp]* and examined whole-brain myelin structures (Fig. S10). Previously, we had established this transgenic zebrafish line [44], in which membrane-targeted GFP is expressed under the control of the *myelin basic protein* (*mbp*) gene promoter. Adult brain tissue clearing and high-resolution imaging uncovered an unknown myelinated bundle structure between CCe and Va. The structures are located bilaterally within the Va. Compared to wild-type siblings, the structure is reduced and disorganized in *znf536* KO zebrafish (Fig. 6D and S11).

## Discussion

Genomic studies have implicated *ZNF536* in neuropsychiatric disorders, particularly schizophrenia [2]. The orthologous zebrafish gene, *znf536*, displays widespread brain expression (Fig. S2A, B), and we found that loss of *znf536* results in numerous behavioral and neuroanatomical phenotypes. Although we initially did not recover adult homozygous KO animals, we discovered that their growth was being inhibited by the inability to compete for food and were able to rescue them by separated raising (Figs. 1 and 2). This approach allowed us to characterize adult animals, expanding our experimental repertoire to include behavioral assays not possible at the larval stage. Both the behavior and brain anatomy of *znf536* KO zebrafish were abnormal compared to siblings, with the cerebellum being particularly affected.

Behavioral impairment is a hallmark of several neuropsychiatric disorders [16, 45, 46] and is observed in multiple zebrafish genetic knockouts for their risk factors. In particular, we found significantly reduced social interaction in *znf536* KO zebrafish (Fig. 4), similar to other known zebrafish neuropsychiatric models [18, 47, 48]. The novel tank diving assay also revealed anxiolytic activity in *znf536* KO zebrafish, similar to a knockout of the *dyrk1aa* ortholog of the *DYRK1A* autism risk gene [16]. Previously, a screen found that larval stage *znf536* KO mutants preferred the center of the well of a 96-well plate [14]; this larval phenotype may be a correlate of these adult behaviors of more relevance to neuropsychiatric disorders. In addition, we found a reduced response to aversive sound stimuli in *znf536* KO zebrafish (Fig. S4), corroborating a possible anxiolytic state [30, 31]. Other interpretations of these results could include a defect in sensory processing or motor coordination, although the mutant’s average locomotion speed at baseline is unchanged (Fig. 3C).

To determine the underlying abnormalities that could lead to the extensive behavioral phenotypes in *znf536* KO zebrafish, we examined the function and anatomy of the KO brains. We observed reduced expression of neuronal activation marker gene *c-fos* in the corpus cerebelli (CCe) after exposure to a novel tank (Fig. 6A, B”’), indicating abnormal neural function. Histological analysis of *znf536* KO zebrafish adult brain revealed a smaller volume of the valvular cerebelli (Va) region, a part of the cerebellum in fish (Fig. 5A–D”’). The change in cerebellum size is at least in part due to a substantial reduction of Purkinje cells (*pvalb7, aldoca, ca8*) in the Va (Fig. 6C). These neural phenotypes are consistent with the observed changes in the behavior of *znf536* KO mutants. For example, dysfunctional cerebellar Purkinje cells can lead to anxiolytic behavior and social impairment in the episodic ataxia 2 murine model [49]. While the Va is considered a sensory-motor control region in fish [50], it may also play a role in the social and anxiolytic changes observed in *znf536* KO zebrafish.

While the cerebellum is classically known to be an important factor for motor function, recent studies suggested its involvement in social cognition [51] and emotional processing [52]. The critical role of the cerebellum in the pathogenesis of schizophrenia has been emphasized by “cognitive dysmetria” theory [52]. Cerebellar abnormalities have been linked to autism-like manifestations in mouse models [53] and several neuropsychiatric disorders, including schizophrenia, in humans [54]. Dysfunctional cerebellar Purkinje cells have also been reported in human and mouse models [55–60]. Recent human neuroimaging studies demonstrating cerebellar involvement in schizophrenia have discovered aberrant functional connectivity of the cerebellar white matter tracts, thalamus, and other cerebral cortical regions [61, 62].

The cerebellum consists of two major structures: the cerebellar cortex and cerebellar nuclei (CN). The cerebellar cortex receives and processes inputs, while the CN sends outputs which route the results of cerebellar computations to the rest of the brain. However, the CN is often overlooked, as most research focuses on the cerebellar cortex [63]. In humans and other mammals, the CN consists of three pairs: the fastigial nucleus, the interposed nucleus, and the dentate nucleus. In zebrafish, however, eurydendroid cells provide cerebellar output, and the existence of a CN-like structure is still unclear [63]. Importantly, for the first time to our knowledge, we identified a unique myelinated structure located bilaterally within valvular cerebelli, which was disrupted in the *znf536* KO zebrafish. Given that the CN is embedded in the white matter of the medullary center in other vertebrates, we suggest this myelinated structure may be a cerebellar peduncle-like structure in zebrafish (Figs. S10 and S11). Although there are anatomical differences between the human and fish brains, there may be conservation of these structures [64].

Taken together, our findings highlight the importance of *znf536* in the development and function of the cerebellum. Although the precise genetic impact of schizophrenia-associated SNPs on *ZNF536* expression or function in humans is unknown, and thus the translational relevance of our zebrafish model remains unclear, understanding genes that impact vertebrate cerebellar development will yield insight into schizophrenia and other disorders. Recently, we also observed a reduction of Va size in other zebrafish models of autism and intellectual disability [16, 65], nominating Va structural abnormality as a suitable biomarker for the study of neuropsychiatric disorders in zebrafish. Our discovery of the myelinated structure embedded in Va suggests that the zebrafish cerebellum could be more similar to that of mammals than previously thought. In the future, it will be important to characterize the cell types within this structure as well as their connectivity and define the underlying molecular mechanisms leading to its disruption in *znf536* KO zebrafish.

### Supplementary information

Supplemental Material

## Data Availability

This study includes no data deposited in public repositories.

## References

[1] Owen MJ, Sawa A (2016). Mortensen pb. Schizophrenia Lancet.

[2] Schizophrenia Working Group of the Psychiatric Genomics Consortium. Biological insights from 108 schizophrenia-associated genetic loci. Nature. 2014;511:421–7. 10.1038/nature13595 PMC4112379 25056061

[3] Trubetskoy V, Pardiñas AF, Qi T, Panagiotaropoulou G, Awasthi S, Bigdeli TB (2022). Mapping genomic loci implicate genes and synaptic biology in schizophrenia. Nature..

[4] Pocklington AJ, Rees E, Walters JT, Han J, Kavanagh DH, Chambert KD (2015). Novel findings from CNVs implicate inhibitory and excitatory signalling complexes in schizophrenia. Neuron.

[5] Singh T, Poterba T, Curtis D, Akil H, Al Eissa M, Barchas JD (2022). Rare coding variants in ten genes confer substantial risk for schizophrenia. Nature..

[6] Alerts GN, Get RS (1963). Effect of chlorpromazine or haloperidol on formation of 3-methoxytyramine and normetanephrine in mouse brain. Acta Pharmacol Toxicol.

[7] Van Rossum JM (1996). The significance of dopamine-receptor blockade for the mechanism of action of neuroleptic drugs. Arch Int Pharmacodyn Ther.

[8] Satterstrom FK, Walters RK, Singh T, Wigdor EM, Lescai F, Demontis D (2019). Autism spectrum disorder and attention deficit hyperactivity disorder have a similar burden of rare protein-truncating variants. Nat Neurosci.

[9] Turner TN, Coe BP, Dickel DE, Hoekzema K, Nelson BJ, Zody MC (2017). Genomic patterns of de novo mutation in simplex autism. Cell.

[10] Winham SJ, Cuellar-Barboza AB, McElroy SL, Oliveros A, Crow S, Colby CL (2014). Bipolar disorder with comorbid binge eating history: a genome-wide association study implicates APOB. J Affect Disord.

[11] Lin E, Kuo PH, Liu YL, Yu YW, Yang AC, Tsai SJ (2018). A deep learning approach for predicting antidepressant response in major depression using clinical and genetic biomarkers. Front Psychiatry.

[12] Dmitrzak-Weglarz M, Paszynska E, Bilska K, Szczesniewska P, Bryl E, Duda J (2021). Common and unique genetic background between attention-deficit/hyperactivity disorder and excessive body weight. Genes.

[13] Qin Z, Ren F, Xu X, Ren Y, Li H, Wang Y (2009). ZNF536, a novel zinc finger protein specifically expressed in the brain, negatively regulates neuron differentiation by repressing retinoic acid-induced gene transcription. Mol Cell Biol.

[14] Thyme SB, Pieper LM, Li EH, Pandey S, Wang Y, Morris NS (2019). Phenotypic landscape of schizophrenia-associated genes defines candidates and their shared functions. Cell.

[15] Kalueff AV, Stewart AM, Gerlai R (2014). Zebrafish as an emerging model for studying complex brain disorders. Trend Pharmacol Sci.

[16] Kim OH, Cho HJ, Han E, Hong TI, Ariyasiri K, Choi JH (2017). Zebrafish knockout of Down syndrome gene, DYRK1A, shows social impairments relevant to autism. Mol Autism.

[17] Choi JH, Jeong YM, Kim S, Lee B, Ariyasiri K, Kim HT (2018). Targeted knockout of a chemokine-like gene increases anxiety and fear responses. Proc Natl Acad Sci USA.

[18] Geng Y, Zhang T, Alonzo IG, Godar SC, Yates C, Pluimer BR (2022). Top2a promotes the development of social behavior via PRC2 and H3K27me3. Sci Adv.

[19] May M, Hwang KS, Miles J, Williams C, Niranjan T, Kahler SG (2015). ZC4H2, an XLID gene, is required for the generation of a specific subset of CNS interneurons. Hum Mol Genet.

[20] Lee YR, Khan K, Armfield-Uhas K, Srikanth S, Thompson NA, Pardo M (2020). Mutations in FAM50A suggest that Armfield XLID syndrome is a spliceosomopathy. Nat Commun.

[21] Lee YR, Kim SH, Ben-Mahmoud A, Kim OH, Choi TI, Lee KH (2021). Eif2b3 mutants recapitulate phenotypes of vanishing white matter disease and validate novel disease alleles in zebrafish. Hum Mol Genet.

[22] Mendes HW, Neelakantan U, Liu Y, Fitzpatrick SE, Chen T, Wu W (2023). High-throughput functional analysis of autism genes in zebrafish identifies convergence in dopaminergic and neuroimmune pathways. Cell Rep.

[23] Geng Y, Peterson RT (2019). The zebrafish subcortical social brain as a model for studying social behavior disorders. Dis Model Mech.

[24] Bae YK, Kani S, Shimizu T, Tanabe K, Nojima H, Kimura Y (2009). Anatomy of zebrafish cerebellum and screen for mutations affecting its development. Dev Biol.

[25] Thisse B, Heyer V, Lux A, Alunni V, Degrave A, Seiliez I, et al. Spatial and temporal expression of the zebrafish genome by large-scale in situ hybridization screening. In: Methods in cell biology. vol. 77. Academic Press; 2004. pp. 505–19 10.1016/s0091-679x(04)77027-2 15602929

[26] Kirshenbaum AP, Chabot E, Gibney N (2019). Startle, pre-pulse sensitization, and habituation in zebrafish. J Neurosci Method.

[27] Strähle U, Scholz S, Geisler R, Greiner P, Hollert H, Rastegar S (2012). Zebrafish embryos as an alternative to animal experiments—a commentary on the definition of the onset of protected life stages in animal welfare regulations. Reprod Toxicol.

[28] Cachat J, Stewart A, Grossman L, Gaikwad S, Kadri F, Chung KM (2010). Measuring behavioral and endocrine responses to novelty stress in adult zebrafish. Nat Protocol.

[29] Egan RJ, Bergner CL, Hart PC, Cachat JM, Canavello PR, Elegante MF (2009). Understanding behavioral and physiological phenotypes of stress and anxiety in zebrafish. Behav Brain Res.

[30] Chanin S, Fryar C, Varga D, Raymond J, Kyzar E, Enriquez J (2012). Assessing startle responses and their habituation in adult zebrafish. Zebrafish Protocol Neurobehav Res.

[31] Ro Y, Noronha M, Mirza B, Ansari R, Gerlai R. The Tapping assay: a simple method to induce fear responses in zebrafish. Behav Res Method 2021;16:1–4. 10.3758/s13428-021-01753-9 34918220

[32] Miller N, Gerlai R (2007). Quantification of shoaling behaviour in zebrafish (Danio rerio). Behav Brain Res.

[33] Wright D, Krause J (2006). Repeated measures of shoaling tendency in zebrafish (Danio rerio) and other small teleost fishes. Nat Protocol.

[34] Buske C, Gerlai R (2011). Shoaling develops with age in Zebrafish (Danio rerio). Progr Neuro-Psychopharmacol Biol Psychiatry.

[35] Gerlai R (2014). Social behavior of zebrafish: from synthetic images to biological mechanisms of shoaling. J Neurosci Methods.

[36] Reyhanian N, Volkova K, Hallgren S, Bollner T, Olsson PE, Olsén H (2011). 17α-Ethinyl estradiol affects anxiety and shoaling behavior in adult male zebra fish (Danio rerio). Aqua Toxicol.

[37] Miller N, Greene K, Dydinski A, Gerlai R (2013). Effects of nicotine and alcohol on zebrafish (Danio rerio) shoaling. Behav Brain Res.

[38] Dreosti E, Lopes G, Kampff AR, Wilson SW (2015). Development of social behavior in young zebrafish. Front Neural Circuit.

[39] Saverino C, Gerlai R (2008). The social zebrafish: behavioural responses to conspecific, heterospecific, and computer animated fish. Behav Brain Res.

[40] Mahabir S, Chatterjee D, Buske C, Gerlai R (2013). Maturation of shoaling in two zebrafish strains: a behavioral and neurochemical analysis. Behav Brain Rese.

[41] Miyamura Y, Nakayasu H (2001). Zonal distribution of purkinje cells in the zebrafish cerebellum: analysis by means of a specific monoclonal antibody. Cell Tissue Res.

[42] Tanabe K, Kani S, Shimizu T, Bae YK, Abe T, Hibi M (2010). Atypical protein kinase C regulates primary dendrite specification of cerebellar purkinje cells by localizing Golgi apparatus. J Neurosci.

[43] Seeman P (2013). Schizophrenia and dopamine receptors. Eur Neuropsychopharmacol.

[44] Jung SH, Kim S, Chung AY, Kim HT, So JH, Ryu J (2010). Visualization of myelination in GFP‐transgenic zebrafish. Dev Dyn.

[45] Bielsky IF, Hu SB, Szegda KL, Westphal H, Young LJ (2004). Profound impairment in social recognition and reduction in anxiety-like behavior in vasopressin V1a receptor knockout mice. Neuropsychopharmacology.

[46] Egashira N, Tanoue A, Matsuda T, Koushi E, Harada S, Takano Y (2007). Impaired social interaction and reduced anxiety-related behaviour in vasopressin V1a receptor knockout mice. Behav Brain Res.

[47] Liu CX, Li CY, Hu CC, Wang Y, Lin J, Jiang YH (2018). CRISPR/Cas9-induced shank3b mutant zebrafish display autism-like behaviors. Mol Autism.

[48] Ruzzo EK, Pérez-Cano L, Jung JY, Wang LK, Kashef-Haghighi D, Hartl C (2019). Inherited and de novo genetic risk for autism impacts shared networks. Cell.

[49] Bohne P, Mourabit DB, Josten M, Mark MD (2021). Cognitive deficits in episodic ataxia type 2 mouse models. Hum Mol Genet.

[50] Russell CJ, Bell CC (1978). Neuronal responses to electrosensory input in mormyrid valvula cerebelli. J Neurophysiol.

[51] Van Overwalle F, Baetens K, Mariën P, Vandekerckhove M (2014). Social cognition and the cerebellum: a meta-analysis of over 350 fMRI studies. Neuroimage.

[52] Schmahmann JD, Guell X, Stoodley CJ, Halko MA (2019). The theory and neuroscience of cerebellar cognition. Ann Rev Neurosci.

[53] Wang SS, Kloth AD, Badura A (2014). The cerebellum, sensitive periods, and autism. Neuron.

[54] Andreasen NC, Pierson R (2008). The role of the cerebellum in schizophrenia. Biol Psychiatry.

[55] Maloku E, Covelo IR, Hanbauer I, Guidotti A, Kadriu B, Hu Q (2010). Lower number of cerebellar purkinje neurons in psychosis is associated with reduced reelin expression. Proc Natl Acad Sci USA.

[56] Tran KD, Smutzer GS, Doty RL, Arnold SE (1998). Reduced purkinje cell size in the cerebellar vermis of elderly patients with schizophrenia. Am J Psychiatr.

[57] Skefos J, Cummings C, Enzer K, Holiday J, Weed K, Levy E (2014). Regional alterations in purkinje cell density in patients with autism. PLoS ONE.

[58] Shevelkin AV, Terrillion CE, Abazyan BN, Kajstura TJ, Jouroukhin YA, Rudow GL (2017). Expression of mutant DISC1 in Purkinje cells increases their spontaneous activity and impairs cognitive and social behaviors in mice. Neurobiol Dis.

[59] Veleanu M, Urrieta-Chávez B, Sigoillot SM, Paul MA, Usardi A, Iyer K (2022). Modified climbing fiber/Purkinje cell synaptic connectivity in the cerebellum of the neonatal phencyclidine model of schizophrenia. Proc Natl Acad Sci USA.

[60] Tsai PT, Hull C, Chu Y, Greene-Colozzi E, Sadowski AR, Leech JM (2012). Autistic-like behaviour and cerebellar dysfunction in Purkinje cell Tsc1 mutant mice. Nature.

[61] Magnotta VA, Adix ML, Caprahan A, Lim K, Gollub R, Andreasen NC (2008). Investigating connectivity between the cerebellum and thalamus in schizophrenia using diffusion tensor tractography: a pilot study. Psychiatry Res Neuroimaging.

[62] Chang X, Jia X, Wang Y, Dong D (2022). Alterations of cerebellar white matter integrity and associations with cognitive impairments in schizophrenia. Front Psychiatry.

[63] Kebschull JM, Casoni F, Consalez GG, Goldowitz D, Hawkes R, Ruigrok TJ, et al. Cerebellum lecture: the cerebellar nuclei—core of the cerebellum. Cerebellum. 2023;13:1–58. 10.1007/s12311-022-01506-0 PMC10951048 36781689

[64] Kebschull JM, Richman EB, Ringach N, Friedmann D, Albarran E, Kolluru SS (2020). Cerebellar nuclei evolved by repeatedly duplicating a conserved cell-type set. Science.

[65] Lee YR, Thomas M, Roychaudhury A, Skinner C, Maconachie G, Crosier M, et al. Eye movement defects in KO zebrafish reveals SRPK3 as a causative gene for an X-linked intellectual disability. Res Sq. 20:rs.3.rs-2683050. 10.21203/rs.3.rs-2683050/v1 (2023)

