# Supplementary material for: Impairments of cerebellar structure and function in a zebrafish KO of neuropsychiatric risk gene znf536
Source: Transl Psychiatry. 2024 Feb 8;14:82. doi: 10.1038/s41398-024-02806-1 (PMC10853220; doi:10.1038/s41398-024-02806-1)
Supplement: Supplementary file 1 — Supplemental Material [file 41398_2024_2806_MOESM1_ESM.pdf]

SUPPLEMENTAL DATA

Impairments of cerebellar structure and function in a zebrafish KO of neuropsychiatric risk gene *znf536*

Tae-Yoon Kim[1,5], Arkaprava Roychaudhury[1,5], Hyun-Taek Kim[2,5], Tae-Ik Choi[1], Seung Tae Baek[3], Summer B. Thyme[4,6], and Cheol-Hee Kim[1,6]

Table S1. Primers used to generate the RNA probes used in this study.

Gene	Forward primer (5′ – 3′)	Reverse primer (5′ – 3′)	Transcript Source
aldoca	AGAACTCGGATGATGGCGTC	GGTTTGACGGGGTAGTCTCG	ENSDART00000080377.6
c-fos	GACAGGATGATGTTTACCAGCCTT	CCTCCTCACTCTTTGAGATTCCAC	NM_205569.1
ca8	GTGGGGCTCAACCCAAACTA	CCTCCTCCACGCTGTTGAAT	ENSDART00000140012.1
crhb	ACAGACGCGCCGCGCAAAGT	GGCTGATGGGTTCGCTTGTGGT	NM_001007379.1
gad1b	CTCTAGAGATGCGGGAGATC	TTACAGATCCTGACCGAGCC	NM_194419.1
grik2	CAGAATCAGCAACATGTTGGGATTTGTCTC	ACAGAGCCAAATCACTTCACTCTTCTGCAC	XM_001923942.6
pvalb7	AACGTGACTCGACGACCAAG	AGACAGAGCCTTCAGTCCCA	ENSDART00000131386.3
th1	AAACCAGACCCAGCCGAAAA	AGCCGCAATGTTTCTCCAGT	ENSDART00000040410
th2	GGAGCCTTTACCCCAGTCAC	GTCCAGCCCCATAAGCCTTT	ENSDART00000162572
znf536	GGTGTATCCTCGGTGGTCTC	AGGGATATTTGCGGTTCTTGC	ENSDART00000170390.3

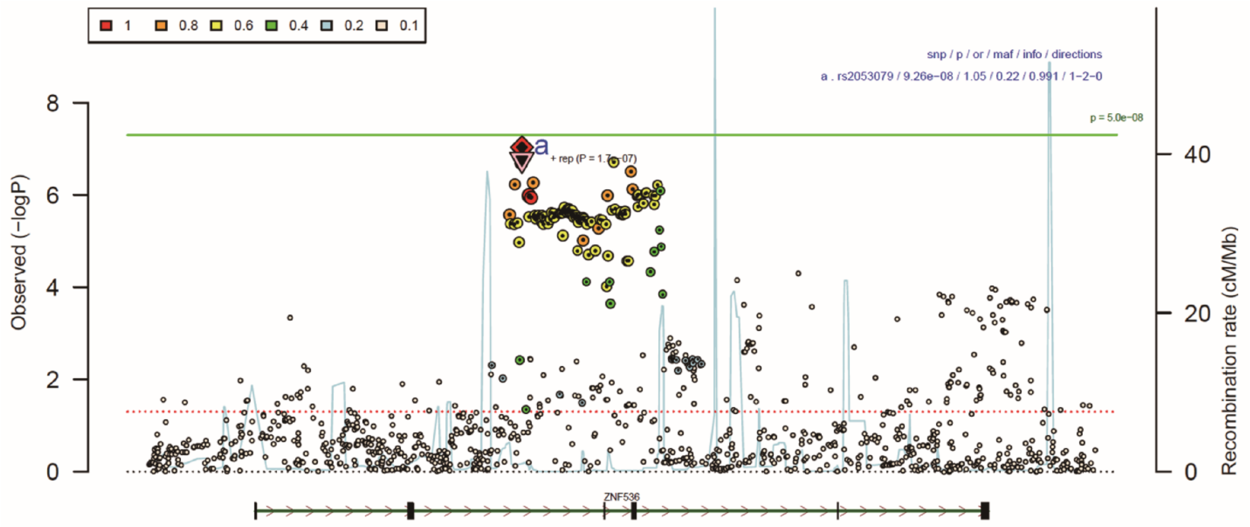

Fig. S1 Ricopili Plot displays the common variant (GWAS) association of *ZNF536* as a candidate risk factor for schizophrenia. The two-sided P values of each SNP from the GWAS meta-analysis are shown along the y axis. The color of each dot corresponds to the linkage disequilibrium with the index SNP, and the properties of the index SNP are displayed [1].

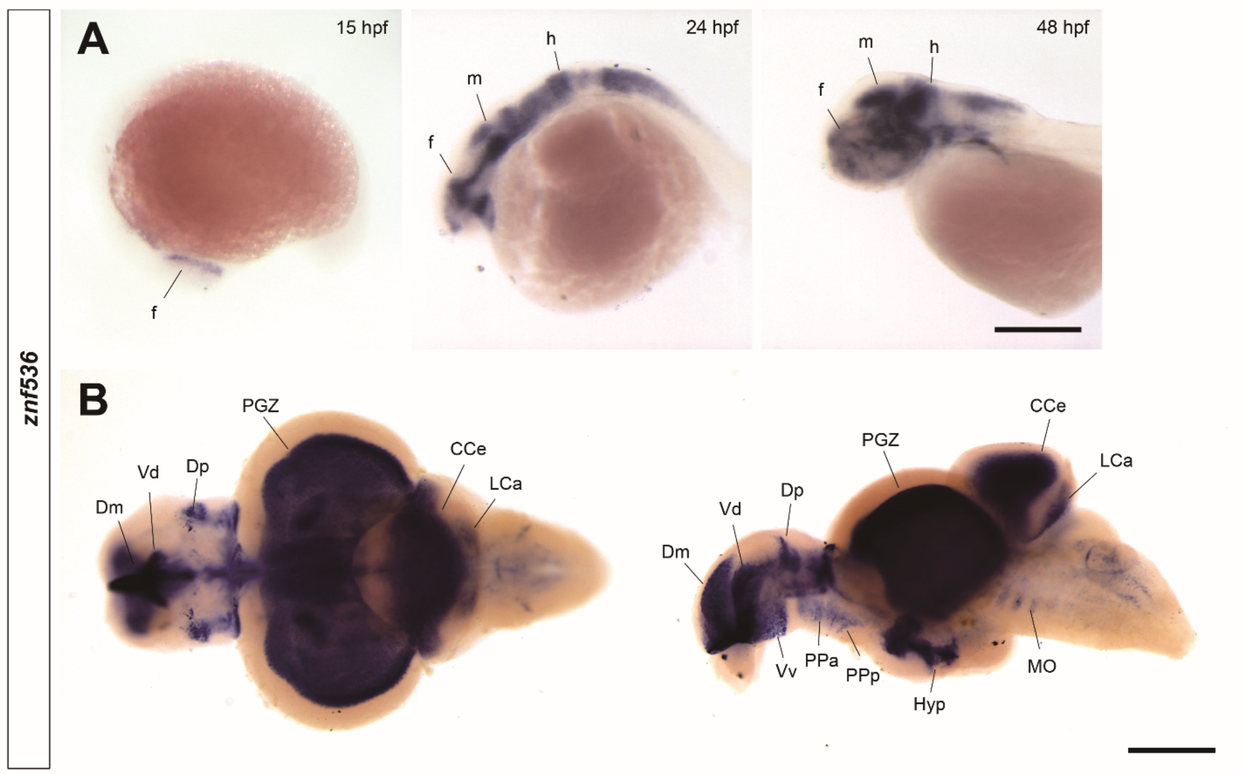

Fig. S2 Spatiotemporal expression of *znf536* mRNA in zebrafish nervous system. A *znf536* expression is first detected ventral forebrain and expands to various brain regions, including the midbrain, hindbrain, and spinal cord. **B** *znf536* expression in an isolated adult brain, showing expression in specific brain regions. Dorsal and lateral view; anterior is to the left. CCe, corpus cerebellum; Dm, medial zone of the dorsal telencephalic area; Dp, posterior zone of the dorsal telencephalic area; f, forebrain; h, hindbrain; Hyp, hypothalamus; LCa, Lobus caudalis; m, midbrain; MO, medulla oblongata; PGZ, periventricular gray zone; PPa, parvocellular preoptic area (anterior part); PPp, parvocellular preoptic area (posterior part); Vd, dorsal zone of ventral telencephalic area; Vv, ventral zone of the ventral telencephalic area. Scale bar: 200 µm (A), 500 µm (B).

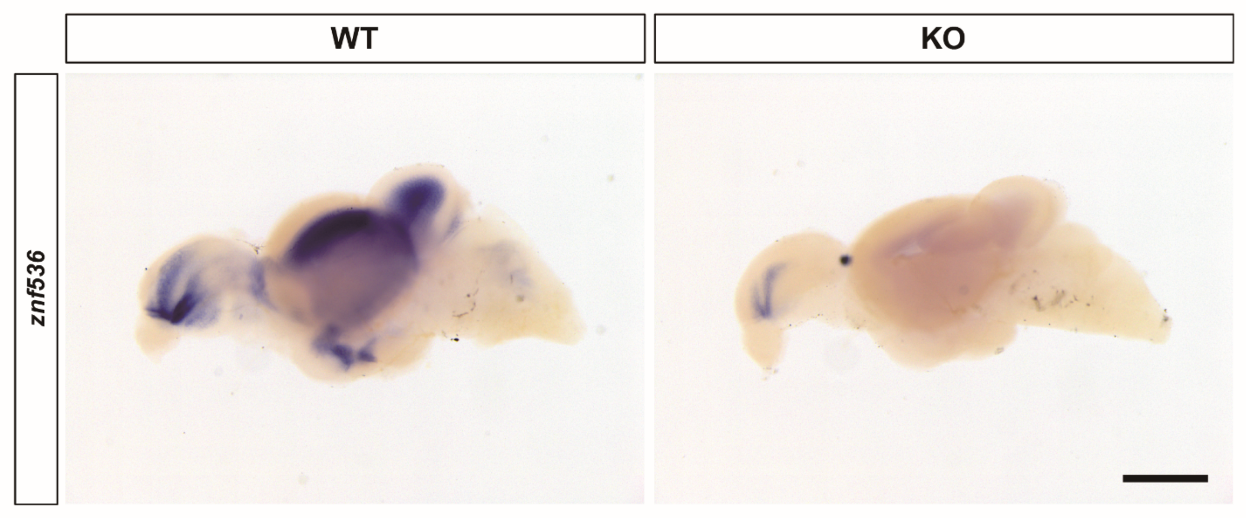

Fig. S3 Comparison of *znf536* mRNA expression between *znf536* KO and WT zebrafish adult brain. Lateral view; anterior is to the left. n = 2 for WT and n = 2 for KO zebrafish. Scale bar: 500 μm.

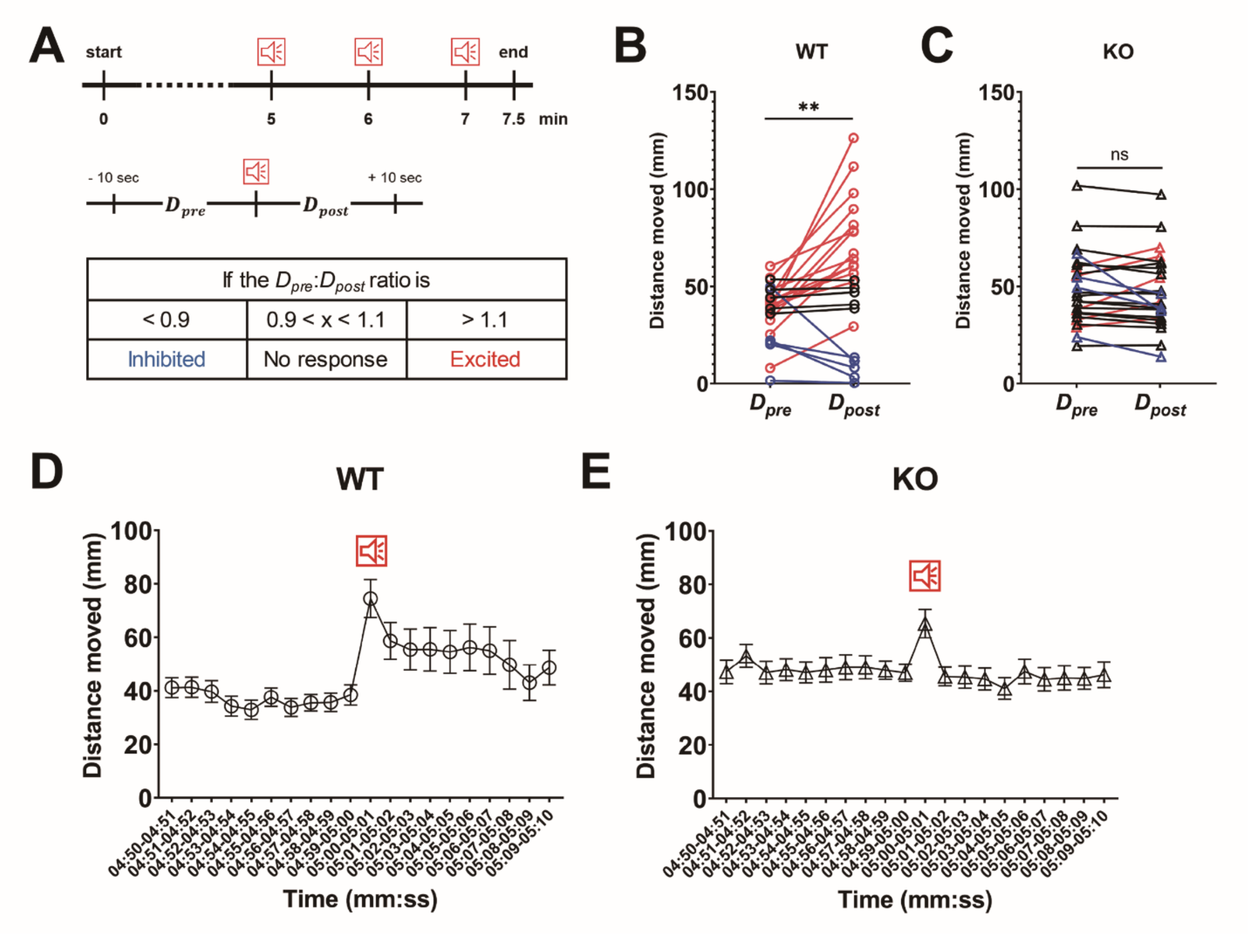

Fig. S4 Acoustic startle response assay in *znf536* KO zebrafish. A Experimental setup for the assay in adult zebrafish. The ratios of distance moved (D) before/after stimulus (post/pre) were grouped into three groups; “Excited”, “No response”, and “Inhibited”. **B**,**C** Pairwise comparison of the distance moved. Red lines, Excited; black, No response; and blue, Inhibited. n = 25 for WT and n = 25 for KO zebrafish. Statistical significance was determined by Wilcoxon signed-rank test (one-tailed). ns, no significance; p**<0.01. **D,E** Distance moved before/after the first startle stimulus. The distance moved was measured every second before and after the stimulus.

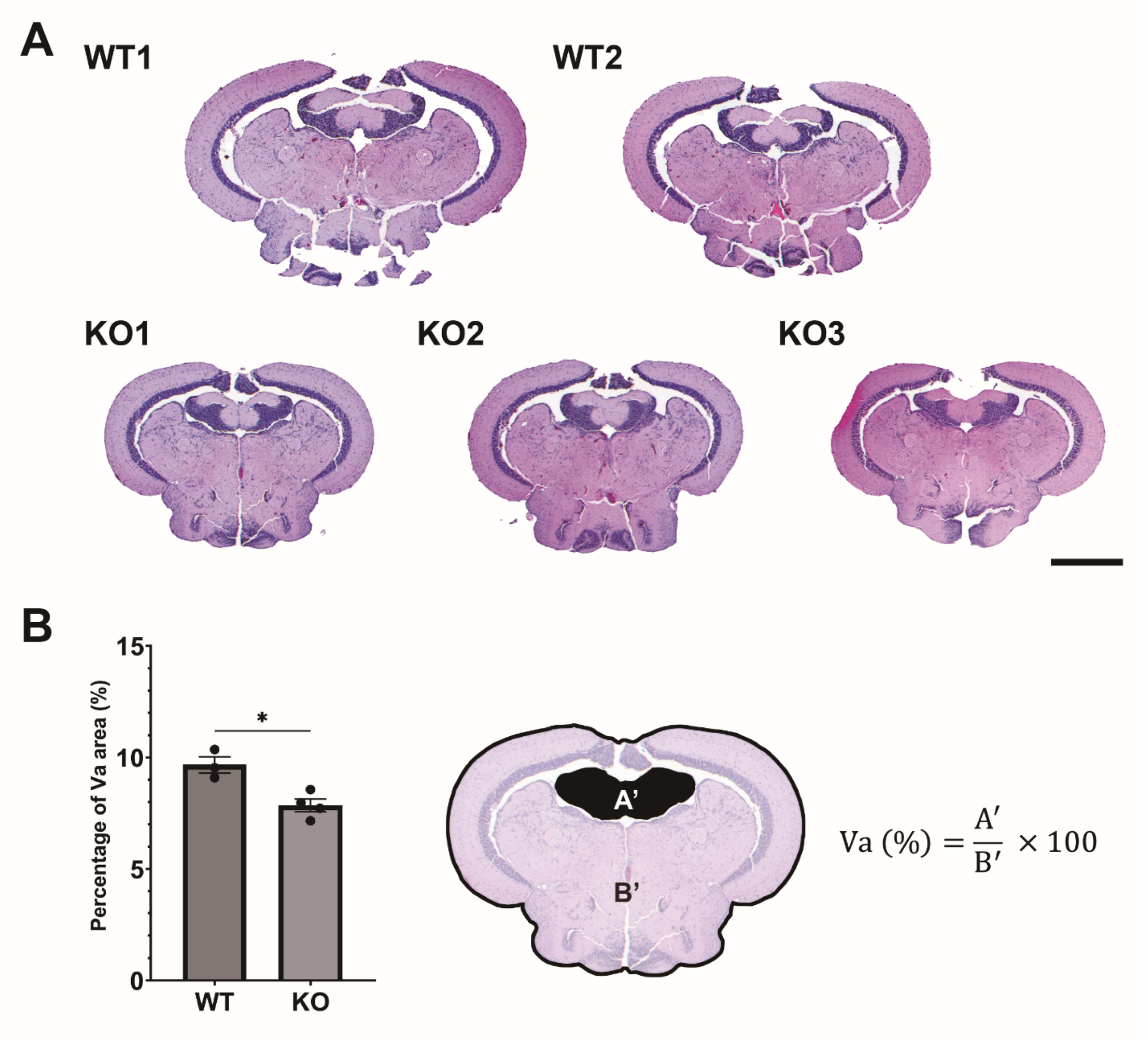

Fig. S5 Histological analysis of adult brains (6 mpf). A Representative brain images at matched sections between wild type (WT1, WT2) and *znf536* KO (KO1, KO2, KO3) zebrafish. Male sibling fish were used for this comparison. Scale bar: 500 μm. **B** Quantified data for percentage of Va area between WT and KO. Percentage of Va area was measured by ImageJ analysis; Va area (A') versus total brain area (B'). n = 3 for WT and n = 4 for KO. Data was presented as mean ± standard error of the mean (S.E.M.). Statistical significance was determined by Mann-Whitney test (one-tailed). p*<0.05.

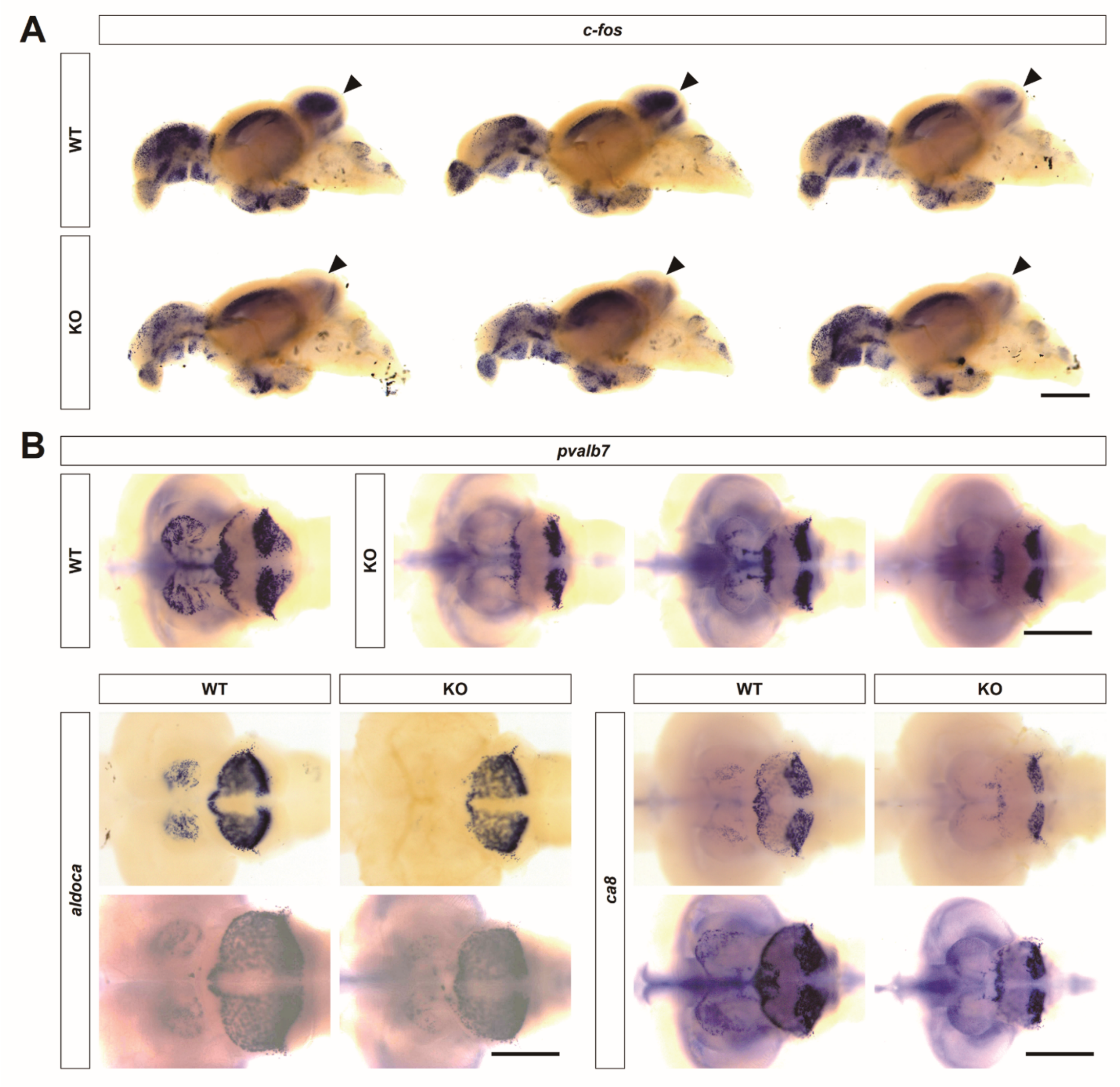

Fig. S6 Additional replicates of neuronal and Purkinje cell markers, related to Figure 6. A Additional replicates of *c-fos* expression after novel tank assay. Arrowheads indicate cerebellum. **B** Additional replicates of Purkinje cell markers, *pvalb7*, *ca8* and *aldoca*. Scale bar: 500 µm.

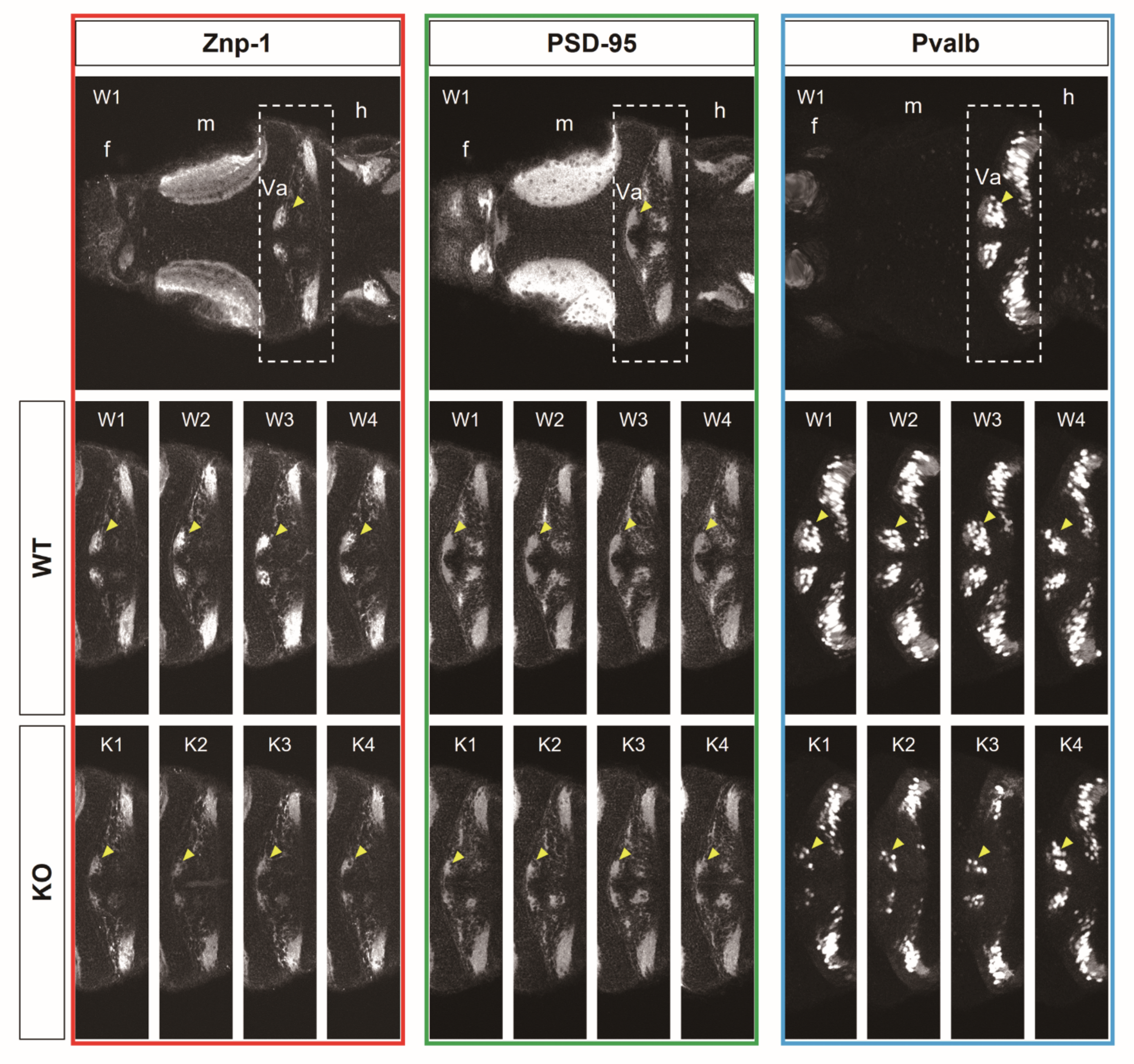

Fig. S7 Decreased expression of neural markers in the cerebellum of *znf536* KO zebrafish at 6 dpf. Detection of specific neuronal types in the developing valvular cerebelli (Va, arrowheads) at 6 dpf of the previously published *znf536*^{a211} KO line, stained with anti-Znp-1 (1/500, znp-1-s, DSHB, Iowa, USA), anti-PSD-95 (1/75, K28/86-S, DSHB, Iowa, USA), or anti-Pvalb (1/500, gift from Dr. Masahiko Hibi) antibodies, respectively. Top panel: a representative animal and selected region of the cerebellum (boxed) from multiple animals in bottom panels. Dorsal views with anterior to the left. f, forebrain; h, hindbrain; m, midbrain; Va, Valvular cerebelli. Whole-mount staining was conducted by standard methods, and the stacks were registered to the Z-Brain standard reference brain using a total-Erk stain (1/500, Cell Signaling CAT#4696) [2]. Dorsal views with anterior to the top. The Pvalb is shown as a maximum intensity projection of slices 90-95 of the 138-slice Z-Brain, while the other two stains are slice 106. Four example fish are shown. Pvalb: n = 6 KO, 6 WT; PSD-95: n = 16 KO, 12 WT; Znp-1: n = 15 KO, 13 WT.

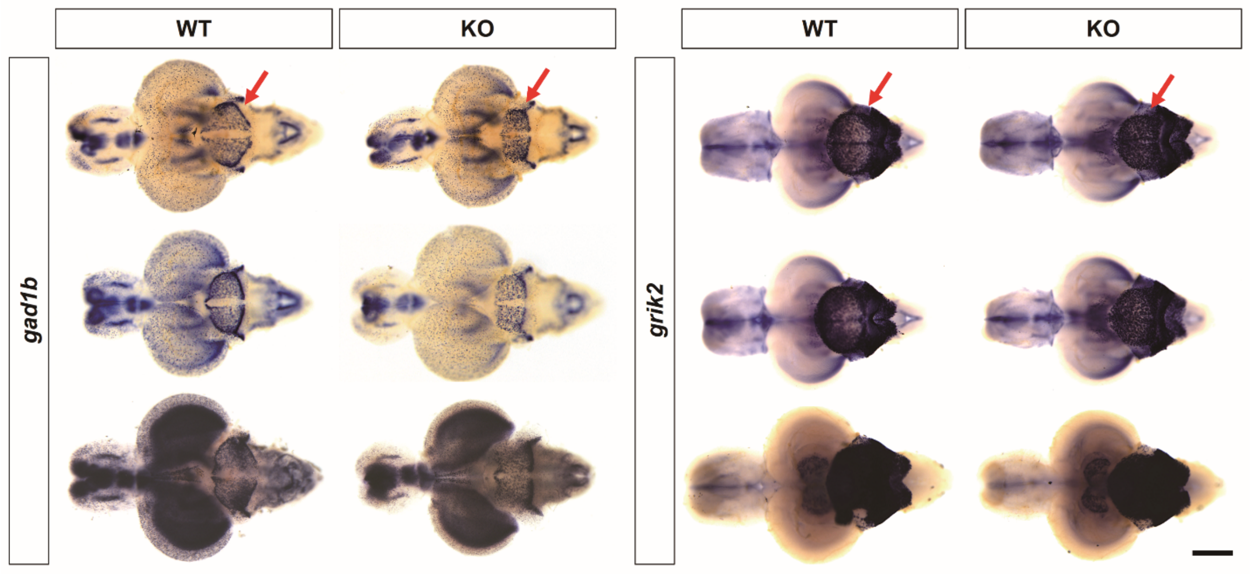

Fig. S8 Decreased expression of neural markers in the cerebellum of adult *znf536* KO zebrafish. Expression of GABAergic neuronal marker (*gad1b*) and Bergmann glia marker (*grik2*) in the adult brain. Dorsal views with anterior to the left. Cerebellum is indicated by arrow. *gad1b*: n = 3 for WT and n = 3 for KO; *grik2*: n = 3 for WT and n = 3 for KO. Scale bar: 500 μm.

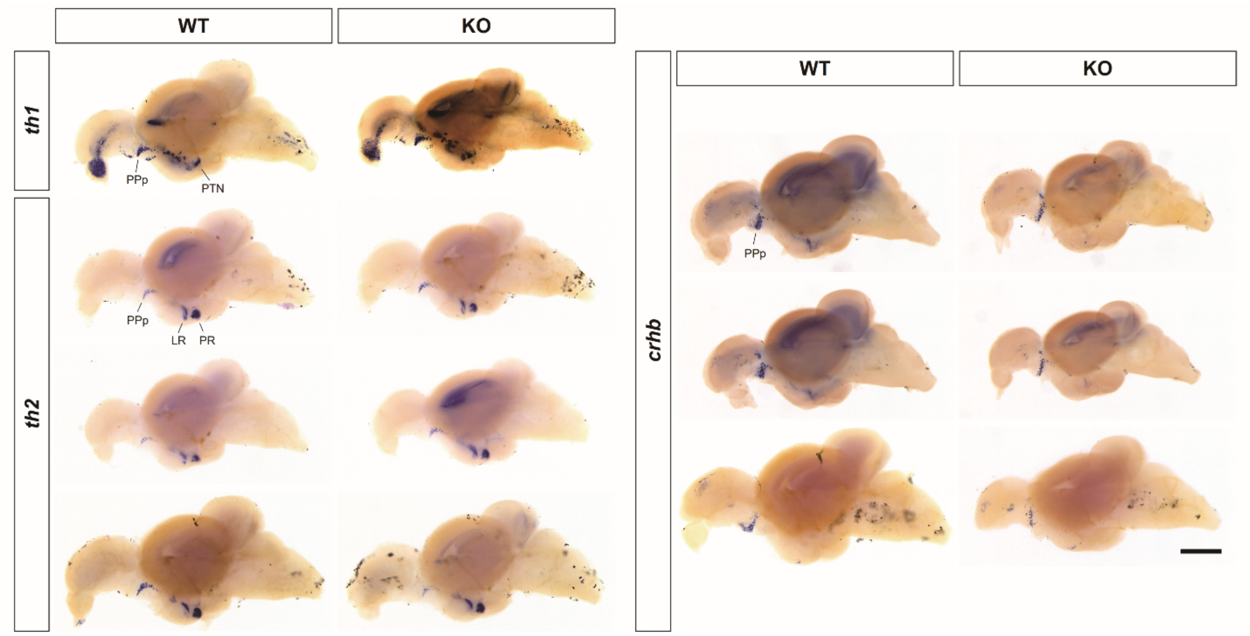

Fig. S9 Expression of dopaminergic neuronal markers (*th1*, *th2*) and stress hormone marker (*crhb*) in KO zebrafish brain. LR, lateral recess of diencephalic ventricle; PPp, parvocellular preoptic nucleus, posterior part; PR, posterior recess of diencephalic ventricle; PTN, posterior tuberal nucleus. Scale bar: 500 μm.

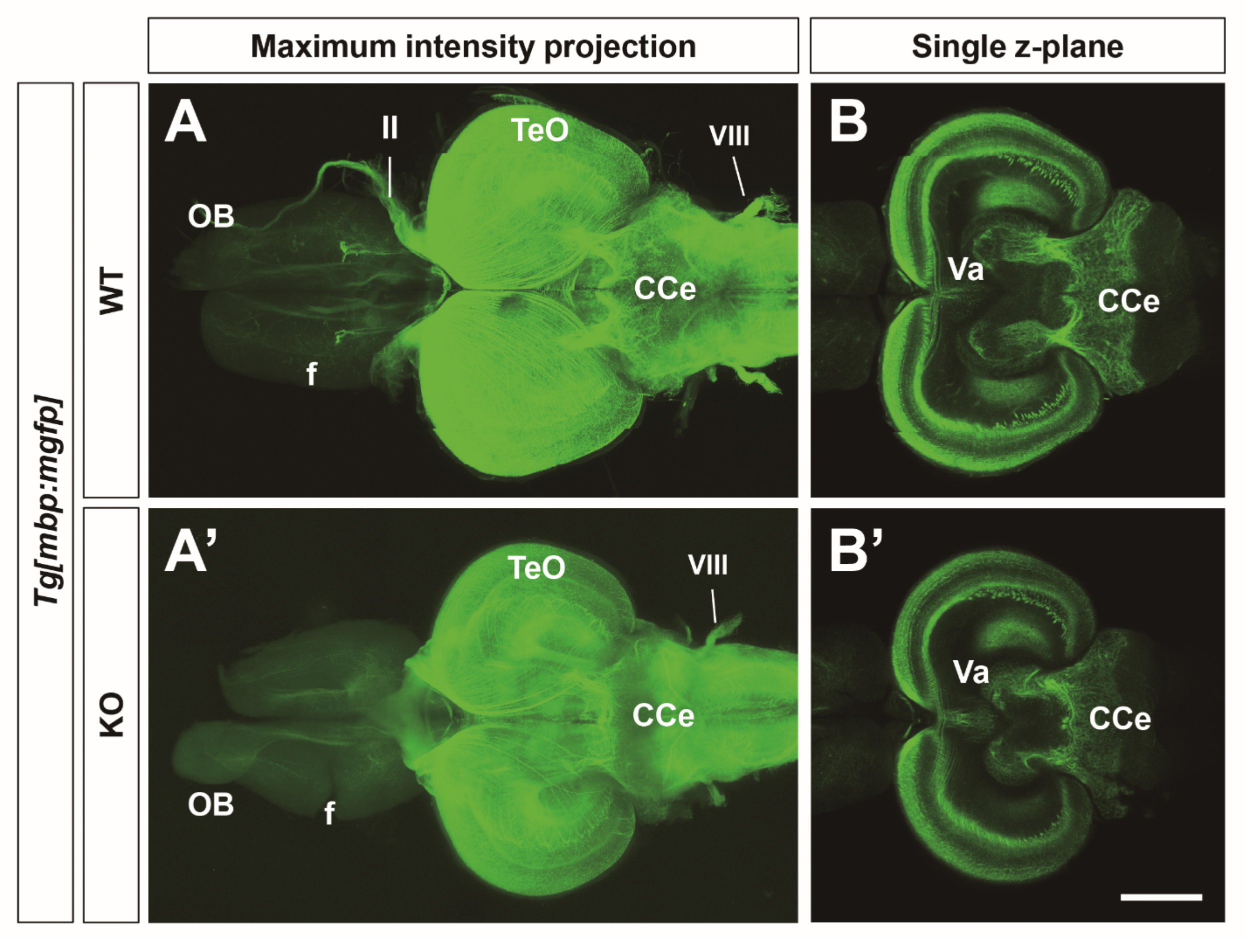

Fig. S10 Confocal microscopy images after tissue clearing. A,A' Maximum intensity projection of adult brain isolated from WT and KO zebrafish crossed with *Tg[mbp:mgfp]*. **B,B'** A single z-plane for visualizing the valvular cerebelli and the corpus cerebelli. n = 2 for WT and n = 2 for KO. CCe. corpus cerebelli; f, forebrain; OB, olfactory bulb; TeO, optic tectum; Va, valvular cerebelli; II, optic nerve; VIII, auditory/octaval nerve. Scale bar: 500 μm.

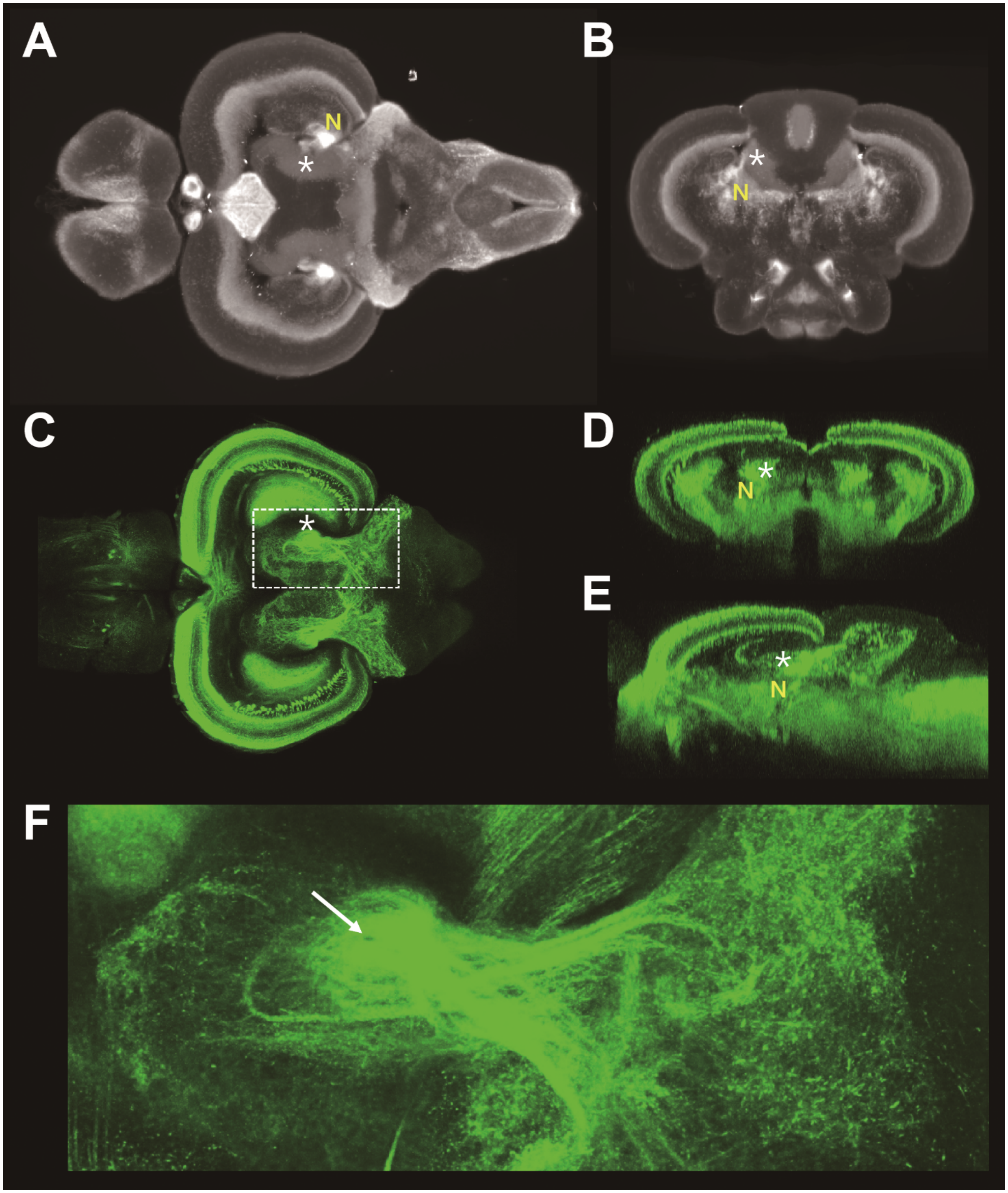

Fig. S11 Anatomical analysis of Va myelin tracts in comparison with zebrafish brain atlas. A,B Whole brain stained with a pan-neuronal marker HuC. Images were obtained from the adult zebrafish brain atlas [3]. **C-E** Myelin tracts in WT adult brain of *Tg[mbp:mgfp]* line. Asterisk indicates a unique structure within the Va region. "N" indicates the nucleus lateralis valvulae (NLV) region, which serves as an anatomical landmark. **A,C** Horizontal optical section. Anterior is to the left. **B,D** Coronal section. Dorsal is to the top. **E** Sagittal section. Anterior is to the left. **F** Enlargement of inlet in **C**, showing a vortex-like organization of myelin bundles with a small hole in the center (arrow).

REFERENCES

1 Trubetskoy V, Pardiñas AF, Qi T, Panagiotaropoulou G, Awasthi S, Bigdeli TB, et al. Mapping genomic loci implicates genes and synaptic biology in schizophrenia. Nature. 2022 Apr 21;604(7906):502-8.

2 Randlett O, Wee CL, Naumann EA, Nnaemeka O, Schoppik D, Fitzgerald JE, et al. Whole-brain activity mapping onto a zebrafish brain atlas. Nat Methods. 2015 Nov;12(11):1039-46.

3 Kenney JW, Steadman PE, Young O, Shi MT, Polanco M, Dubaishi S, et al. A 3D adult zebrafish brain atlas (AZBA) for the digital age. Elife. 2021 Nov 22;10:e69988.
